# Self-Flagellation as Possible Route of Human T-Cell Lymphotropic Virus Type 1 Transmission

**DOI:** 10.3201/eid2510.190484

**Published:** 2019-10

**Authors:** Claire E. Styles, Veronica C. Hoad, Paula Denham-Ricks, Dianne Brown, Clive R. Seed

**Affiliations:** Australian Red Cross Blood Service, Perth, Western Australia, Australia (C.E. Styles, V.C. Hoad, C.R. Seed);; Australian Red Cross Blood Service, Melbourne, Victoria, Australia (P. Denham-Ricks, D. Brown)

**Keywords:** Human T-lymphotropic virus type 1, blood donors, risk factors, self-injurious behavior, viruses, Australia

**To the Editor:** Blood donors in Australia who test positive for transfusion-transmissible infections, including human T-lymphotropic virus (HTLV), hepatitis B virus (HBV), hepatitis C virus, and HIV, undergo posttest counseling, as previously described (*1*). Similar to Tang et al. (*2*), we identified self-flagellation as a possible unique risk factor for HTLV-1 infection. History of self-flagellation was elicited in 7 (28%) of 25 HTLV-1–positive donors identified during January 2012–December 2018. All 7 donors were men 20–37 years of age, of whom 5 were born in Pakistan and 2 in India; 6 had given blood in Victoria, Australia. The 18 remaining HTLV-1–positive donors were 29–68 years of age; 10 (56%) were men; 1 was born in India and none in Pakistan; and 7 (39%) gave blood in Victoria.

HBV shares transmission routes with HTLV-1 and is highly infectious, including through minor blood exposures (*3*). After discussion of recognized infective risk factors, the 610 HBV-positive donors from the same period, of whom 83 were born in India or Pakistan, were asked about any other potential blood exposures. None reported self-flagellation.

At the time of posttest counseling, no previous HTLV results were available for donors reporting self-flagellation or for their family members. Until the known modes of vertical and sexual transmission have been excluded by such results, the likelihood of self-flagellation as an infective risk factor remains unclear. Although India and Pakistan are not known to be geographic risk areas for HTLV-1, few prevalence studies are available (*4*), and HTLV-1 is commonly present in small geographic foci (*5*). In addition, a noticeable degree of transmission through communal self-flagellation would first require a raised prevalence of infection among the practicing group. We look forward to further research that may clarify the apparent link between self-flagellation and HTLV-1 infection.
